# From Fragmentation to Integration: Enhancing Staff Awareness and Signposting Capabilities via a Collaborative Digital Resource Directory

**DOI:** 10.1192/bjo.2026.11845

**Published:** 2026-06-30

**Authors:** Viyakhulen Sooriyakumaran, Ayse Ozcelik, Nikhil Dhir

**Affiliations:** South West London and St Georges’ NHS Trust, London, United Kingdom

## Abstract

**Aims:**

The purpose of this audit was to evaluate and improve staff awareness and confidence in signposting patients to services both within and outside the NHS. It aimed to transition from a fragmented information landscape to an integrated “one-stop-shop” directory. By centralising resources into a sustainable, living database, the project sought to bridge knowledge gaps, foster Trust-wide collaboration, and ensure patients receive tailored support across all five boroughs.

**Methods:**

A Trust-wide baseline survey was distributed to all staff, capturing data on professional roles and geographical boroughs to identify specific resource gaps. Targeted surveys were sent to medical staff to pinpoint clinical areas and service types where signposting confidence was lowest. Both quantitative and qualitative methods were used to gather initial baseline data and further suggestions to integrate resources better. This data informed the design of a Microsoft Lists database, prioritizing the most “under-resourced” areas and ensuring the platform met the practical needs of frontline staff.

**Results:**

Baseline data confirmed a high demand for resources, with 71.5% of staff signposting patients at least weekly. Key barriers included unclear referral pathways (77.8%), poor service awareness (70.4%), and outdated information (59.3%). Pre-intervention, staff lacked confidence in signposting for housing (78.7%), employment (67%), and finances (57.1%), with significant unawareness regarding legal aid (89.3%) and food banks (67.9%). Internal service literacy was also low, with over half (60.7%) unaware of Trust rehabilitation services. Consequently, 60.7% of staff requested the interactive digital directory.

Post-intervention, confidence in signposting to employment, housing, and finance services all rose to above 56%. Staff reported marked increases in awareness for domestic abuse and carer support (both 93.8%), legal aid (75%), and debt advice (68.8%). While awareness of external resources improved, gaps persisted for internal rehabilitation (62.5% unaware) and forensic services (43.8% unaware). Qualitative feedback confirmed the directory successfully consolidated information, though staff recommended expanding the database through closer collaboration with community teams and social prescribers across all five boroughs.

**Conclusion:**

The collaborative digital directory represents a low-cost, high-impact solution to streamline patient signposting. Future interventions will focus on expanding the project team and recruiting “resource champions” from various teams to ensure the directory remains dynamic and sustainable. This model serves as a scalable blueprint for enhancing health literacy of staff for services that exist both within and outside NHS services.

